# Exploring the potential of village community banking as a community-based financing system for house improvements and malaria vector control in rural Tanzania

**DOI:** 10.1371/journal.pgph.0002395

**Published:** 2023-11-03

**Authors:** Winifrida P. Mponzi, Dickson S. Msaky, Peter Binyaruka, Emmanuel W. Kaindoa

**Affiliations:** 1 Environmental Health and Ecological Sciences Department, Ifakara Health Institute, Ifakara, Tanzania; 2 The Nelson Mandela, African Institution of Science and Technology, School of Life Sciences and Bio Engineering, Tengeru, Arusha, United Republic of Tanzania; 3 Department of Health System, Impact Evaluation and Policy, Ifakara Health Institute, Dar es Salaam, Tanzania; 4 School of Pathology, Faculty of Health Sciences, University of the Witwatersrand and the Centre for Emerging Zoonotic and Parasitic Diseases, National Institute for Communicable Diseases, Johannesburg, South Africa; University of Oxford, UNITED KINGDOM

## Abstract

House improvement is associated with remarkable reductions in indoor mosquito bites and disease incidences, even in typical rural houses. However, its exploitation remains extremely poor in Tanzania and other endemic countries due to limited financial resources. Nevertheless, village community banks (VICOBA), practiced in Tanzania for nearly two decades, have proven to provide financial services to rural communities that would otherwise not be able to get them from formal financial institutions. This study explored the need, opinion, and willingness of VICOBA members to use VICOBA platforms as a source of finance for improving local houses and eventually controlling mosquito-borne diseases. A mixed-methods approach was used in this study, whereby a survey was administered to 150 participants and twelve focus group discussions were done in three villages in Ulanga district, rural Tanzania. The FGDs comprised eight participants each, with equal representation of males and females. The FGD guide was used to probe the opinions of study participants on malaria transmission, housing condition improvements, and financial resources. About 99% of all participants indicated the urgent need to improve their houses to prevent mosquito bites and were willing to utilize VICOBA for improving their houses. In the focus group discussion, the majority of people who participated were also in need of improving their houses. All participants confirmed that they were at the highest risk of getting mosquito-borne diseases, and they were willing to use money that was either saved or borrowed from their VICOBA for housing improvements and vector control. A self-sustaining financial system destined for house improvement and related interventions against malaria and other mosquito-borne diseases is crucial. The community members were willing to use VICOBA as a source of finance for house improvement and disease control; however, there was limited knowledge and sensitization on how they could utilize VICOBA for disease control.

## Background

Over the past decade, malaria mortality has decreased from 897,000 deaths in the year 2000 to 577,000 deaths in 2019 [1], though in 2020 there were increase in both malaria cases and deaths due to disruption of health systems caused by COVID-19 [2]. Malaria deaths declined slightly, from 625,000 in 2020 to 619,000 in 2021; this decline was associated with the initiatives that were taken by malaria stakeholders [1] to rescue the situation that happened during the COVID-19 pandemic [3]. The overall decline of malaria cases and deaths was due to the use of interventions such as LLINs [4, 5], indoor residual spray (IRS) [6, 7], early diagnosis with malaria rapid diagnostic tests (MRDT), and treatment. However, malaria and other mosquito-borne diseases are still the leading causes of morbidity and mortality, especially for children under five years in low- and middle-income countries [2]. Previous studies demonstrated that most malaria and other mosquito-borne diseases transmission predominantly occurs indoors [8]. Unfortunately, the primary vector control tools (LLINs and IRS), which are for indoor use only, are facing challenges associated with insecticide resistance [9, 10], which puts a large population that lives in sub-Saharan Africa (SSA) at a very high risk of getting malaria and other mosquito-borne diseases. Hence the need for other complementary interventions to fight against malaria and other mosquito-borne diseases.

House improvement refers to screening of openings such as doors, windows, and open eaves, as well as tightening of unfitted doors [11]. House improvement has been demonstrated to be an option for providing protection for malaria and other mosquito-borne diseases transmitted indoors [12–14]. Previous studies demonstrated that blocking open eaves, tightening windows and doors, and putting netting materials in windows (house screening) reduces the mosquito density inside the houses [15–20] for example, in the USA, due to better housing, malaria and other mosquito-borne diseases have been eliminated [21]. A multi-country analysis of 29 malaria surveys from 21 countries in SSA associated modern houses with a lower incidence of malaria, with an effect similar to LLINs [15]. Reductions in indoor mosquitoes have been shown in typical rural houses after slight modifications [14, 15, 22]. Also, thatched-roofed houses with closed eaves and well-fitted doors showed over 94% fewer malaria vectors [15]. Screening of windows and doors also reduced non-malarial vectors indoors by 89–93% [12, 20, 23]. Moreover, the shift from mud walls and thatched roofs to brick walls and tin roofs has been suggested to significantly reduce disease transmission risk. Another study demonstrated that in rural Tanzania, where most houses were mud houses, the mosquito density was very high indoors [24]. These houses are poorly constructed with open eaves, unscreened windows and doors, and unfitted windows and doors, which are the main entry points for mosquitoes. Hence, house improvement can also be used to complement the primary vector control tools available since it can also help fight against insecticide resistance since it doesn’t require any insecticides.

Significant evidence exists for house improvements and the reduction of malaria transmission in many settings; however, most communities are unable to improve their houses due to financial constraints [24]. The evidence also shows the communities need to improve their houses for not only disease control but also to improve the quality of their lives [25, 26]. In most cases, high expenses for construction materials are the major hindrance [25, 27]. Furthermore, there are limitations due to a lack of financial support from the government and other financial aid for supporting health interventions, especially housing improvements. There are also limited intersectoral efforts or investments between the health, financial, and housing sectors in many African settings, such that the housing and settlement sectors are not well linked with the health sector. The majority of Tanzania’s population, about 66 percent, lives in rural areas and does not have access to financial services from formal financial institutions [28], which they could utilize for housing improvements and eventually to control mosquito-borne diseases. One of the potential approaches to overcome financial constraints is the adoption of village community banking systems (VICOBA), which have proven to improve livelihoods in rural settings [29, 30].

Village community banks (VICOBA) are a form of informal microfinance institution based on small self-helping groups of low-income community members. The group members normally save and share the financial resources. Also, VICOBA is a designed to help individuals seek capital for developing businesses and other personal needs. They are programmed for saving and crediting to empower individuals who need financial services such as loans and savings. The group members must always come from the same village and be known to every member of the group, and they must have a common interest in formulating that particular group. The main goal of the group is to help each other financially so as to fulfill every individual member’s needs within a specific period of time. Usually, the group membership ranges from 10 to 30 members of both genders, depending on the purpose and agreement of the group. The number of group members usually varies from group to group.

A village community banking group is formulated after all group members agree to be involved in the group, and then the group is registered with the local authority. After registration, the first meeting of the group members must first select the group leaders (chairman or woman, secretary, and treasurer). After selecting the group leader, the group members formulate the constitution of the group, which consists of the group’s bylaws, rules, and regulations. The group members have to read and sign that they have read and understood the constitution, and are ready for its implementation. This helps to make every group member accountable in the event that one of the group members defaults on a loan. The group member usually selects specific days of meeting, usually it’s either weekly, biweekly, or monthly, depending on the agreement of the group. But in rural settings, most groups meet weekly to simplify the collection of repayment and savings and also provide loans. Also, the group members use the meetings to discuss about the development of the group and group members in general.

In every meeting, group members have to first contribute savings, which is mandatory. Savings usually range from 5,000 Tshs to 100,000/ = Tshs per month, and every member must pay that amount. Second, for those who have a loan, they have to pay their repayment. Usually repayment can be paid in two different ways: first, if someone takes out a loan for three months, they can pay monthly interest for two months, and in the third month, they can pay the total amount borrowed. Second the loan plus interest can be divided into three months, and that amount can be paid every month. In case a group member fails to pay repayment, she or he has to pay a fine, usually 10% of the total repayment. The group member must request the loan prior to the next meeting, register the names and the amount, and identify two people within the group who will guarantee them. The loans must be assessed and approved by the group members during the group meeting, depending on the member’s capacity to pay. Guarantors will be liable in the event that he or she defaults; the guarantors must repay the loan through their savings, and all of the defaulter’s savings will be used to repay the loan and the remaining balance the guarantors have to repay.

Members are normally allowed to get loans and repay them at an interest rate of 10%, which is taken as income for their group. For example, for a member to borrow 100,000/ = Tshs, he or she must first have savings of not less than 34,000/ = and will be required to repay a total of 110,000 Tshs either within three months or six months, depending on the suggested repayment terms, but most groups pay within three months. Though there are other VICOBA that 10% interest rate is paid monthly, for example, if someone takes a loan of 100,000/ = Tshs, they are supposed to return a total of 130,000/ = Tshs within three months. In the first case, that person will pay a repayment of 37,000Tshs every month, if it’s weekly the repayment will be 9,250 Tshs. In the second case, that person will be supposed to pay a total of 44,000/ = Tshs every month, and if its weekly repayment is 11,000 Tshs. VICOBA slightly differs especially in terms of how the interest rate is calculated, but in other aspects, such as how they give loan and return the operations are the same. The interest that group members contributed by taking the loan will be returned to the members as the profit that they made within one year. The group round lasts for twelve months, during which the group members have to repay all of their loans and interest, and they calculate the total amount that the group has earned throughout the year. After the calculations for those who paid all of their loans they will be given back the amount of savings they invested over a period of one year. For those who didn’t finish their loan, the amount of savings they invested over a period of one year is normally used to pay the loan. After the completion of the first round of one year, the same day they start the next round. This has been and is still in areas where access to financial resources has been challenged. VICOBA have been reported to benefit poorer men and women in developing countries since their establishment [31]. VICOBA does not only save people from rural settings but also from urban communities [32]. For example, in Tanzania, about 27% of the community members who would otherwise not be able to access formal financial institutions are served by VICOBA [28].

As Tanzania and other developing countries work towards achieving Sustainable Development Goal (SDG) 1 (poverty reduction), SDG 3 (Good health and well-being), and SDG 8 (improving economic growth), VICOBA provides opportunities to achieve these goals. One of the major hindrances to economic growth and improvements in health, especially in rural areas, is limited financial resources. In Tanzania, for example, where VICOBA has been practiced mostly in rural areas for nearly a decade now, studies have indicated that VICOBA has a great impact on livelihood and recommend integration with public health [32]. In most cases, VICOBA serves community members to get finance for agriculture, education costs, ceremonies, buying foods [33], starting up small businesses, and house improvements on a limited scale [31]. This study describes the current engagement of VICOBA members and explores the need, opinions, and willingness of VICOBA members to use their VICOBA platform as a potential source of finance for improving local houses and eventually controlling mosquito-borne diseases. Specifically, this study assessed the community’s knowledge about malaria transmission and its association with house characteristics, as well as the need and willingness to use VICOBA for house improvement.

## Methodology

### Ethics statement

Ethical approval for this study was obtained from Ifakara Health Institute Institutional Review Board (IHI/IRB/No: 25–2019) and National Institute of Medical Research (NIMR) through the Medical Research Coordinating Committee (MRCC), Ref: NIMR/HQ/R.8a/Vol. IX/3351. Before conducting the study, we conducted meeting with village leaders in all study villages to explain the aim of the study. Because this study was a purposive study focused with the community member who are the member of VICOBA, Additionally, consent to conduct the study was sought both at the communal and individual level. Communal consent was from face to face discussion with local leaders about the study and requested to conduct it in their village. Individual consent was by discussing with each participant about the study procedures and its importance, followed by a request to participate. For those who agreed to participate were given a written consent forms to sign. Permission to publish this manuscript was obtained from National Institute of Medical Research (Ref: NIMR/HQ/P.12 VOL XXXIII/78).

### Study area

**Fig 1:**
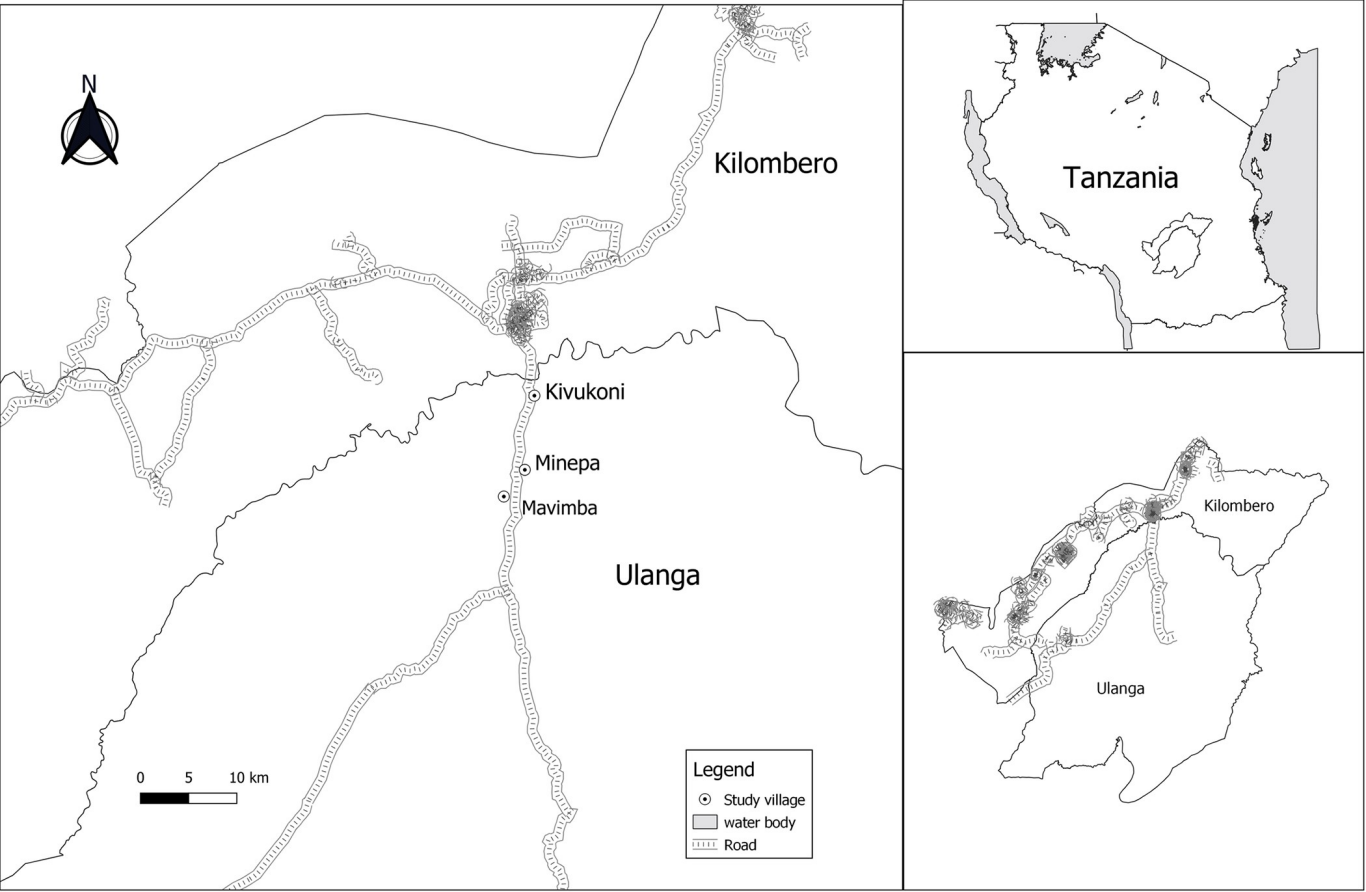
Map of the study area, showing the villages in Ulanga district where the study was conducted. Base map Shapefile Source: The Humanitarian Data Exchange, https://data.humdata.org/dataset/cod-ab-tza?.

**Fig 2:**
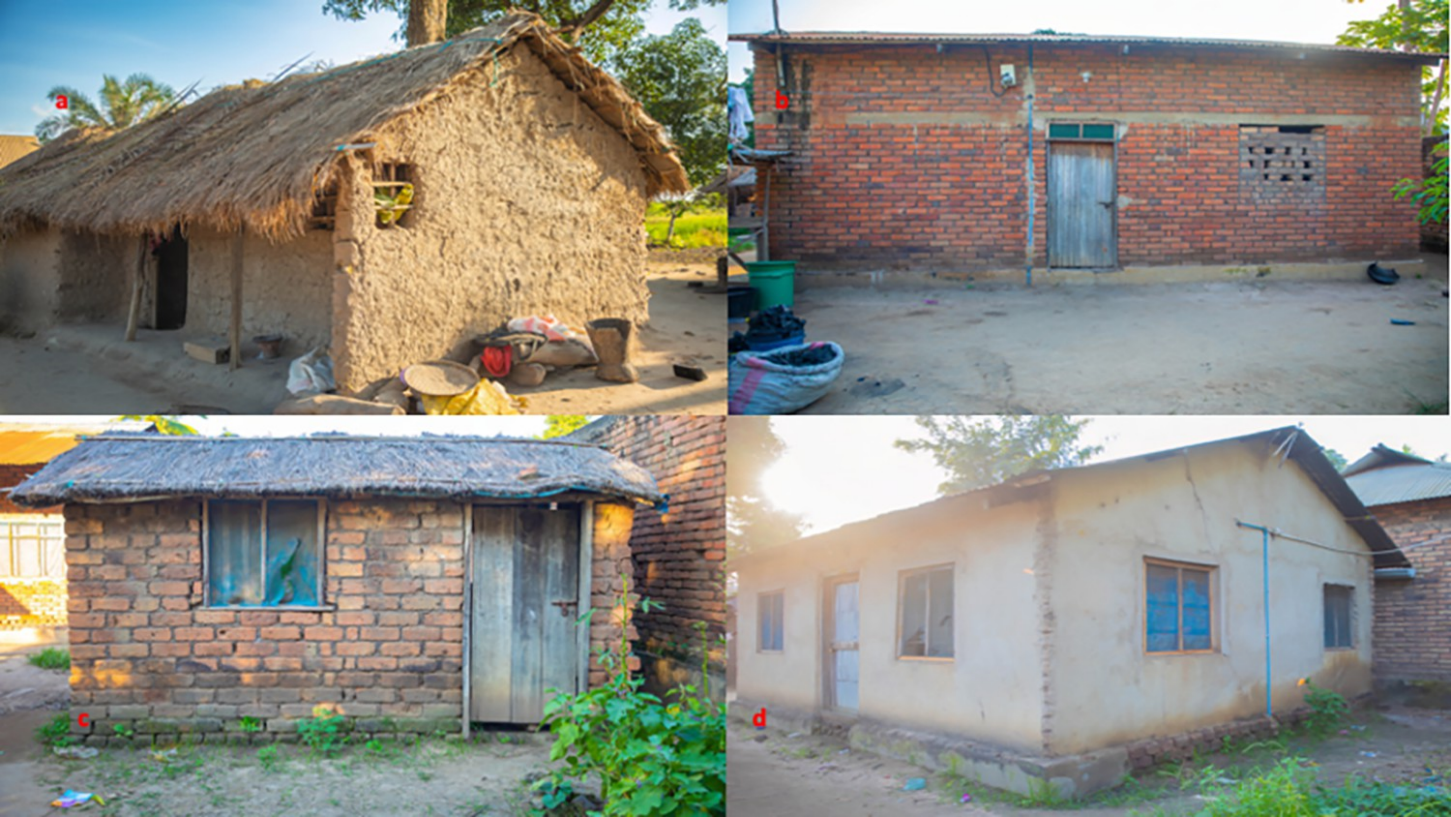
Pictorial representation of typical local house types in the study area: (a) a house with grass thatch roofing with mud walls; (b) a house with corrugated iron roof and brick walls; (c) grass thatch roof with brick walls; and (d) a house with iron sheet roofing and plastered brick walls. Photos by the author.

The study was conducted in the three villages of Kivukoni (8.11°S and 36.42°E), Minepa (8.27°S and 36.67°E) and Mavimba (8.31°S and 36.67°E) in Ulanga district, Morogoro region, south-eastern Tanzania (Fig 1) [34]. These villages lie between altitude of 120–350 meters above sea level, with an annual rainfall and temperature range of 1200-1800mm and 20-38°C respectively [35]. The main economic activity in this area is subsistence rice cultivation [36]. The primary criteria for selecting these villages were: 1) the presence of a well-established and functioning VICOBA system for a very long time; 2) the local authorities have been helping these VICOBA, especially those based in agriculture, by crediting them with fertilizers and paddy seeds; and 3) the majority of residents in these villages live in houses that allow mosquitoes to enter due to the open eaves, unfitted doors, and unscreened windows, which pose a great risk of malaria transmission [24, 37] (Fig 2). These villages have two seasons of rice cultivation, during the wet season as well as during the dry season, where they use irrigation systems [35]; hence, the presence of high mosquito density throughout the year due to the presence of mosquito breeding habitat, which conveys a higher risk of malaria transmission throughout the year. The main malaria-dominant vectors in these villages are *Anopheles arabiensis* and *Anopheles funestus*. The major malaria intervention in these villages is LLINs [38].

### Study design

This study was a descriptive cross-sectional mixed-methods study where a survey was conducted to assess the acceptability and willingness of VICOBA members to use their VICOBA credits for housing improvement. Also, focus group discussions were conducted to encourage people to share their opinions on their needs and willingness to participate in improving their homes using the money they get from their VICOBA platform. The study used two components, including: (i) a semi-structured questionnaire that was administered between January and February 2021 to assess the need and willingness of village community banking members to consider the potential of using the VICOBA system for house improvements and the control of mosquito-borne diseases. A total of 150 VICOBA members were randomly selected from different VICOBA groups in three villages: Kivukoni, Minepa, and Mavimba. Members from different groups aged above 18 years participated in the study, including (ii) focus group discussions to assess people’s knowledge, perceptions, and views on using the VICOBA platform for housing improvement and the control of mosquito-borne diseases. Detailed explanations of each component are provided below.

### Study population

This study identified 27 VICOBA groups from Kivukoni, Minepa, and Mavimba from the village offices. From the list of VICOBA, this study identified only the active groups since the list consisted of active and inactive groups. After having the list of active groups, the randomization was done using an Excel sheet. The study found that, there were 489 active members of VICOBA from both villages. This study considered only group members who were the owners of houses with the help of group leaders. The study managed to identify 372 group members who owned houses and 309 group members whose houses either had open eaves, unscreened windows and openings, or untightened doors.

### Sample size

This was a mixed-methods study where surveys and FGDs were conducted. A survey was conducted to assess the community’s need and willingness to use the VICOBA platform as the source of funds for housing improvement. The criteria for participating in the survey were that one must have an age above 18 years, be an active member of VICOBA, own a house, and the house need to be improved. Since the study had the population of VICOBA, sample size calculation was based on the following formula:

n = N/1+Ne^2^

n = 309/1+309x0.05^2^

n = 174

N = Population

e = Margin error

n = sample size

### Participants recruitment

The participants in this study were selected purposefully because the study needed to assess community members who were members of VICOBA. The randomization was done by listing the names of the VICOBA group in an Excel sheet and randomizing. At the end, we obtained the names of 15 VICOBA members, of whom 174 were selected to administer a questionnaire and 96 FGD. During questionnaire administration, only 150 members participated; the remaining were not found due to farming activities. The participants were visited while conducting their meetings in their specific villages. They were provided with information about the aim of the study and asked if they would be willing to participate in it. At the end of the meetings, 150 VICOBA members participated in the survey (administered the questionnaire), and 96 members participated in the FGD. The number of participants was equally distributed since the number of VICOBA in these villages was approximately the same and the villages were homogenous; they had the same characteristics.

### Survey questionnaire

**Fig 3:**
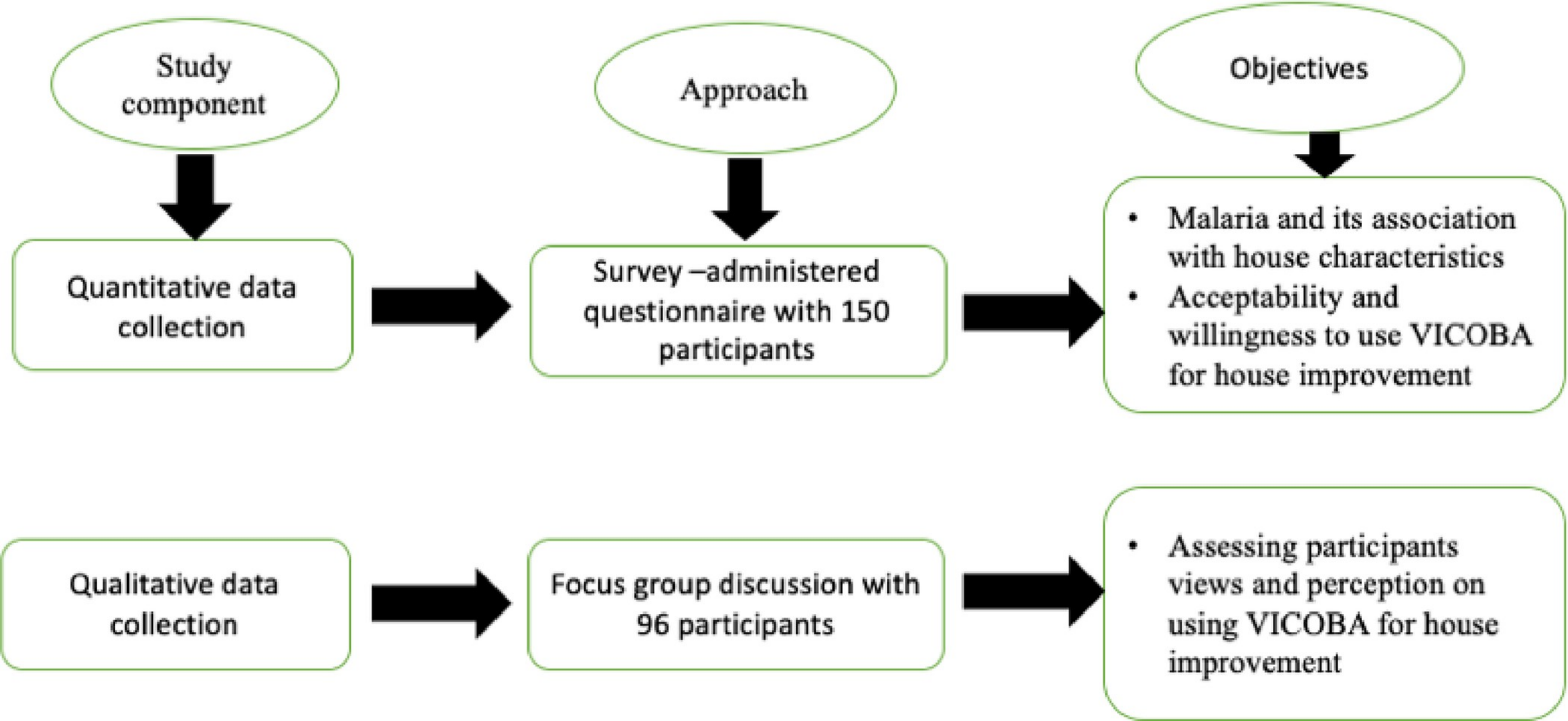
The main themes developed from the survey.

The semi-structured questionnaire had four parts; the first part captured the socio-demographic traits of study participants such as gender, age, occupation, education level, and marital status. The second part captured socio-economic indicators. The third part assessed the mosquito prevention methods that the household member is currently using, while the last part assessed the need and willingness to use VICOBA for improving houses for mosquito prevention. The questionnaire was first given to 10 different potential participants for piloting and was revised accordingly. The major themes captured in the survey are shown in (Fig 3).

### Focus group discussion

The study used a multi-stage sampling approach, first using purposive sampling, whereby only VICOBA groups were selected from the community, and then using a random sampling approach, whereby participants were randomly selected from the VICOBA members. The participants’ ages ranged from (21 to 67 years old). There were separate groups for male and female participants in order to assess their needs and willingness to use VICOBA for house improvements for mosquito protection. Twelve focus group discussions were conducted with a total of 96 representatives (8 participants in each group). The study guide was developed and piloted. The themes in the guideline were generated from the structured questionnaire. The discussion was done in Swahili, the local language, and was recorded using a voice recorder. Additionally, the notes were taken during the discussion by researchers. The FGD sessions took 48–55 minutes. Sessions were conducted in village offices and primary school classrooms.

### Data analysis

#### Analysis of quantitative data

All data were entered and cleaned using Microsoft Excel, and descriptive analysis was performed to capture the descriptive statistics of the following information: (a) the socioeconomic status of study participants and characteristics of households, (b) knowledge and perception of malaria transmission risks in relation to house characteristics; (c) need, willingness, and potential of using VICOBA for house improvement and mosquito control.

#### Processing and analysis of qualitative data

Audio data from FGD was transcribed, and because Swahili was used during the discussion, the data was translated into English. Before analysis, the data was checked to see if it was well translated and documented. The data were analysed using the computer software package for qualitative analysis (Nvivo software version 13). This study used thematic analysis to analyse the collected data. The codebook was developed using inductive coding methods. The main themes developed during the analysis were: mosquito entry points, mosquito problems in the community, the need for housing improvement, and the willingness to borrow money from VICOBA for housing improvement.

## Results

### Socio-demographic traits of participants

Table 1 presents the descriptive results of the socio-demographic traits of the participants. A total of 246 VICOBA members were selected to participate in this study, of which 150 participated in the survey and 96 in the FGD. Sixty-nine percent of VICOBA members who participated in the survey were women, and 31% were men. The majority of participants who participated in the survey had primary and secondary education, and their average age was 37 years, ranging between 20 and 71 years old. About three-quarters of the respondents were farmers (65%), while 27% were businessmen. Two-thirds (78%) were married, 14% were not married, 5% divorced, and 3% were widowed. Also, 65% of the participant’s monthly income ranged from TSHs 100,000/ = to 300,000/ = while 22% of the participant’s monthly income was less than TSHs 100,000/ = (S1 Data).

**Table 1:** Socio-demographic and economic traits of participants.

Variables	N (%)
Gender	
Male	47(31)
Female	103(69)
Age group	
Below 30 Years	42(28)
Above 30 Years	108(72)
Marital status	
Married/Cohabited	117(78)
Single	21(14)
Widowed	4(3)
Divorced/separated	8(5)
Educational status	
Primary	102(68)
Secondary and above	42(28)
Higher	6(4)
Main occupation	
Farmer	97(65)
Business	41(27)
Other	12(8)
Household assets	
Simple mobile phone	131(87)
Radio	102(68)
Bicycle	150(100)
Television	66(44)
Refrigerator	29(19)
Electric fan	36(24)
Electric iron	30(20)
Computer	2(1)
Motorcycle	23(15)
Car	2(1)
House ownerships	
Yes	150(100)
No	-
Monthly earning (Tshs*)	
Less than 100,000	33(22)
100,00–300,000	97(65)
300,000–500,000	17(11)
Greater than 500,000	3(2)
Household size	
1–3 people	49(33)
4–6 people	77(51)
Above 6 people	24(16)

Values are reported as N (%)

Amount in Tanzanian Shillings (1usd = 2300Tshs)

With regards to the FGD, this comprised sixteen groups, of which eight were male and eight were female participants. Their average age was 40 years, ranging between 20 and 67 years; 50% were female participants and 50% were male participants.

### Household characteristics

Table 2 presents the socioeconomic status and house characteristics. Half of participants (57%) reported living in houses with open eaves, while 43% had closed eaves. Fifty-seven percent reported their windows being screened, while 43% had unscreened windows at all, and 33% of the screened windows had some holes. Also, 73% of the participant’s houses confirmed their doors were not fitted well, while 27% of the house’s doors fitted well. Regarding wall types, 48% of participants reported houses had brick walls and concrete, 39% had cement, and 13% had mud walls. Also, 87% reported living in houses with iron roofs, while 13% had thatched roofs (S1 Data).

**Table 2:** House characteristics.

Variable	Response	N (%)
House have opened or closed eaves	Open	85(57)
	Closed	65(43)
House have screened windows	Yes	86(57)
	No	64(43)
The quality of screened windows	Intact screening	36(24)
	Screens with some holes	50(33)
	No screening	64(43)
House door properly fitted	Yes	40(27)
	No	110(73)
Wall type	Bricks and concrete	72(48)
	Cement	58(39)
	Mud	19(13)
	Other	1(0)
Roof type	Zink/Iron/Alluminium sheets	131(87)
	Grass/papyrus/banana leaves	19(13)

Values are reported as N (%)

### Knowledge about malaria transmission and its association with house characteristics

The participants reported that the major entry point for mosquitoes is open eaves (48%), followed by doors (29%) and windows (23%). Fifty-nine percent of the participants were concerned with a very high mosquito problem in their houses, 29% moderate, and 11% low. Regarding how often the participants are bitten by mosquitoes, the majority of participants (67% reported they had been bitten by mosquitoes many times), 25% reported only sometimes, and 7% reported they experienced mosquito bites all the time. Also, 59% of participants reported being bitten by mosquitoes both outdoors and indoors, while 37% reported they experienced mosquito bites only outdoors and 4% indoors. Also, regarding mosquito protection methods, the study found that 90% of the study participants use mosquito nets, 3% do not use any protection, 4% use topical repellent, and 3% use insecticide spray as shown in (Table 3) and (S1 Data).

**Table 3:** Knowledge about malaria transmission and its association with household characteristics.

Variable	Response	N (%)
Mosquito entrance point	Doors	43(29)
	Open eaves	72(48)
	Window	35(23)
Mosquito problem in the house	Low	17 (11)
	Moderate	44 (29)
	High	89(59)
How often bitten by mosquito	Only sometimes	38(25)
	Many times,	101(67)
	All the time	11(7)
Location at home where mosquitoes bite the most	Outdoor	56(37)
	Indoor	6(4)
	Both outdoor and indoor	88(59)
Protection method	Bed net	135(90)
	No protection	4(3)
	Topical repellent	6(4)
	Insecticides spray	4(3)
	Other	1(0)

Values are reported as N (%)

### Mosquito entrance points in houses

The majority of participants reported that mosquitoes use different openings to enter their houses. They mentioned that due to financial constraints, most of their houses are not fully constructed; they have eaves openings, unscreened windows, unfinished windows, and unfitted doors, which allow mosquitoes to enter their houses. They also mention that in the evenings, when they forget to close their doors and windows, they may find a lot of mosquitoes inside the houses.

“*Mosquitoes like to enter areas with openings like unscreened windows and windows with holes*, *some of our houses have a lot of cracks that allow mosquitoes to penetrate inside*. *Also*, *our doors are not in good shape; this might be the reason why we have a large mosquito density in our houses*. *In short*, *we are at a higher risk of getting malaria”*. (34 years old female Mavimba village).

“*The only place where mosquitoes can enter our homes are those open areas*. *Look outside there*, *do you see that opening between roof and wall*? *Most of our houses have that opening*, *in my opinion*, *I think that is the place where mosquitoes use to enter our houses”*. (32 years old male, Kivukoni village).

#### Mosquito problem in the community

Another theme that rose during the discussion was mosquito density inside and outside their houses. They reported that there are a large number of mosquitoes in their houses due to the structure of their houses. They also said most of their houses do not have electricity, and when there is darkness, mosquitoes hide in the darkness, and bite them when they go to sleep or when they sit inside in the evenings and nights.

“*In my house*, *there are a lot of mosquitoes because my house is not fully constructed*, *my windows are not closed*, *my doors are not fitted well*, *and there is an opening between the roof and the wall*, *which I believe contributes a lot to the mosquito population I see inside my house*. *In the evening*, *mosquitoes are very active this is a real challenge to us”*. (41 years old female, Minepa).

“*I don’t have electricity in my house when the sun set in the evening due to the structure of my house you will find a lot of mosquitoes entering my house because it always dark in my house you will find them hiding somewhere*, *and when you sit inside you will find them biting you*. *We have children in our houses these are the ones at a very high risk since they don’t know how to deal with mosquitoes we real need some help”*. (43 years old Male, Mavimba).

### Need and willingness to use VICOBA for housing improvements and malaria control

With regards to the use of the VICOBA platform, the majority of participants (32% reported that they have 1–2 years of experience in VICOBA), 28% have 3–5 years of experience, 25% have greater than seven years of experience, and 11% have 5–7 years of experience. Also, 94% reported having borrowed money from VICOBA, and 6% didn’t borrow money from VICOBA. Regarding how much they save in their VICOBA, 61% save amounts ranging from TSHs 10,000 to 30,0000, 34% save 30,000 to 50,000, 4% save greater than 50,000 and 1% save less than 10,000. Likewise, they reported the amount they borrowed from their VICOBA, 63% borrowed amounts ranging from TSHs 100,000 to 300,000, 16% borrowed from TSHs 300,000 to 500,000, 8% borrowed greater than TSHs 500,000 and 7% borrowed less than TSHs 100,000. Also, they reported the use of the money they borrowed from VICOBA, 51% used the money for agriculture, 16% for businesses, 12% for paying school fees, very few for building homes, and 6% didn’t borrow money but instead used VICOBA as a platform for saving. Furthermore, they reported the issue of repayment, whereby 91% managed to repay their loans while 3% did not manage to repay their loans due to financial problems.

**Fig 4:**
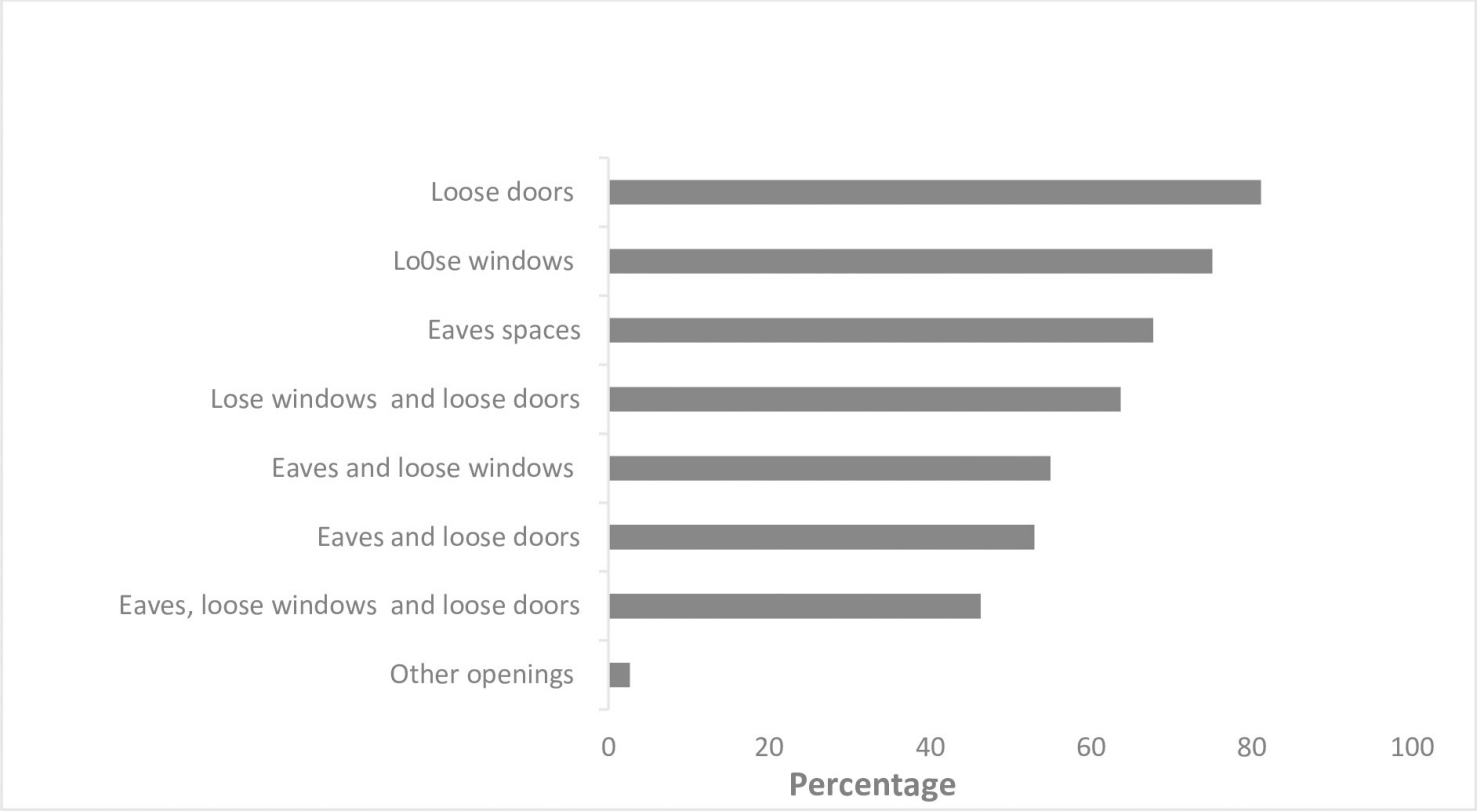
Percentages of the mosquito entry points in the houses that need improvement and willing to use VICOBA for improving the houses.

Moreover, 94% were aware that improving houses can reduce mosquito density inside their houses, and 99% indicated the need to improve their house. Out of 99% of those who need housing improvements, some need to fix their loose doors as mosquito entry points, some need to fix loose windows, some eave spaces, other loose windows and loose door, eave spaces and loose doors both (eaves spaces, loose window and loose door) and some need to fix other openings (Fig 4). Regarding their willingness to borrow money from their VICOBA for improving their house, 99% of the above-mentioned houses were willing to borrow money from their VICOBA to improve their houses for protection against mosquito bites, while among them, 5% are currently using money from VICOBA for house improvement. The summary is shown in (Table 4), (Fig 4) and (S1 Data).

**Table 4:** Knowledge and willingness to use VICOBA for housing improvements and malaria control.

Variable	Response	(N) %
Years current VICOBA	Less than a year	3(2)
	1–3 years	35(23)
	3–5 years	37(25)
	5–7 years	26(17)
	Greater than 7 years	48(32)
	I don’t remember	1(1)
Years in VICOBA	Less than a year	6(4)
	1–3 years	48(32)
	3–5 years	42(28)
	5–7 years	17(11)
	Greater than 7 years	37(25)
Experience in borrowing money from VICOBA	Yes	141(94)
	No	9(6)
Saving in your current VICOBA (Tshs*)/meeting	Less than 10,000	1(1)
	10,000–30,000	92(61)
	30,000–50,000	51(34)
	Greater than 50,000	6(4)
Amount borrowed currently (Tshs*)	Less than 100,000	10(7)
	100,000–300,000	94(63)
	300,000–500,000	25(16)
	Greater than 500,000	12(8)
	I didn’t borrow	9(6)
Uses for the money borrowed from VICOBA	Agriculture	76(51)
	Building home	8(5)
	Business	24(16)
	Purchasing furniture	2(1)
	School fees	19(12)
	I didn’t borrow	9(6)
	Other	12(8)
Managing to repay	Yes	137(91)
	No	4(3)
	I didn’t borrow	9(6)
Repayment terms	3 months	94(63)
	4 months	4(3)
	5 months	2(1)
	6 months	31(21)
	12 months	6(4)
	No	13(8)
Aware that proper house screening prevent mosquito from entering your house	Yes	141(94)
	No	9(6)
Need to improve your house	Yes	149(99)
	No	1(1)
Willing to borrow money from VICOBA for house improvement	Yes	149(99)
	No	1(1)

Values are reported as N (%)

Amount in Tanzanian Shillings (1usd = 2300Tshs)

#### Need to improve houses

Participants had a great desire to renovate their houses and prevent mosquito entry. The majority of the participants wish to renovate their houses, and they suggested that they are at a very high risk of getting malaria and other mosquito-borne diseases. They also added that the major constraint to their delay in renovating their houses was the issue of financial and family priorities. When they get money, they usually think of sending their children to school, using it for farming purposes, and other family issues.

“*Actually*, *this is a problem for many of our houses; if you go around our village*, *you will find that most of our houses are unfished houses; that’s why malaria is still present in our settings*. *We have an irrigation system here*, *so mosquitoes will always be here in our compounds*. *We really need to improve our houses so as to protect not only from mosquito bites but also from other dangerous insects that enter our houses using these openings*.” (37-year-old male, Minepa)

“*I want to have a better house because if you have a better house*, *you will have full assurance that you will not be able to get mosquitoes inside your house*. *I didn’t think that leaving my house the way it is could be the source of all these mosquitoes*. *I think now we need to be serious and make house renovation our priority because even if you pay school fees for your child and your child ends up being sick every day*, *this has no meaning at all”*. (67-year-old male, Kivukoni).

“*You know if you say you have to wait by saving money so as to improve your house when you get enough money*, *it’s a lie; money doesn’t stay*, *and that’s our biggest problem*, *and as we’ve seen*, *you can only fix the areas that have openings so as to block mosquitoes from entering the houses and stop the transmission*. *Some houses need major improvements and others need minor improvements*, *so this can possibly be solved by loans from VICOBA”*. *(*55-year-old female, Mavimba).

#### Willingness to use money they borrow or save from VICOBA for house improvement

We also wanted to know if they would be willing to use their savings or the money they borrow from their VICOBA to renovate their houses for mosquito prevention. The majority reported that they would be willing to borrow money from their VICOBA or use their savings to renovate their houses. They mentioned that they always thought that they needed a lot of money to finish their houses, but even house screening can help them reduce mosquito density in their houses.

“*I will definitely borrow money from VICOBA to renovate my house*. *I think it’s just a matter of priority*. *I have been borrowing money for so long to send my children to school and use money for agricultural purposes*, *but now it’s time to finish my house so we can protect ourselves from mosquito bites*.*” (42 years old*, *female Minepa)*.

“*Ooh*, *yes*, *I will accept the money I borrow or the savings I usually save in our VICOBA to be used for renovating my house*. *I never thought that living in an unfinished house could cause problems like this*. *We never took the issue of renovating houses so seriously; that’s why 90% of our houses are unfinished*. *We need now to educate our fellow community members about house improvement so we can be a mosquito-free zone”* (62-year-old male Kivukoni).

“I *am ready to borrow money from VICOBA to be able to improve my house because I have been thinking about this for a long time*, *and to be honest*, *this issue is only possible if we are serious*. *You know*, *we never thought of even screening our houses; we always thought about rebuilding our houses and making them beautiful like those houses in towns*, *but now we can first think on screens to protect ourselves against the biting*. *Here there are people; we have more than one VICOBA*, *and each one has its own purpose*, *so it is possible that we can establish VICOBAs specifically for housing improvements”* (41-year-old female Mavimba).

#### Challenges facing VICOBA members

This study also explored the challenges that VICOBA members face in their daily operations. It was reported that the main challenges were a lack of capital, with most of the VICOBA members contributing a very small amount of savings in every meeting, which forced them to borrow a small amount of money. Poor repayment records: some members do not return the money in three months as stated in their constitution; instead, they return after six or twelve months, which leads to hardship in borrowing. The other challenge was the lack of financial education for all members of VICOBA, which led to misuse of the money they borrowed.

“*The biggest challenge that we face is lack of capital*, *we really need financial assistance*, *whether from financial institutions or NGOs if possible*. *Having financial assistance will boost our lending capacity*. *We need to borrow money that can possibly change our lives”* (61-year-old male Kivukoni).

“*Honestly*, *all of us lack financial education; if we get financial education*, *we will end up successful”*. (47-year-old woman, Mavimba).

“*Hmmm*, *the challenge we have is that there are a few people who are not good repayers; they always bother to repay; for example*, *the constitution says that if you borrow*, *you should repay all your loan within three months*, *but these people become troublesome and do not follow the procedure*. *Sometimes the guarantors have to bear the costs*, *and sometimes they have to sell the property of that member to pay the debt*. *These are in our groups*, *though they are very few*, *but we cannot know them by looking”* (36-year-old female Minepa).

“*Another challenge is that people save very little; that’s why they cannot borrow money that can solve their problems and hence misuse the funds*. *I think that financial education should be given*, *especially when we start the implementation of this program of improving houses using VICOBA*, *so that those loans can help meet the goals*.*”* (42-year-old female, Mavimba).

## Discussion

The effectiveness of long-lasting insecticidal nets (LLINs) and indoor residual spraying (IRS), which have greatly reduced the malaria burden, is constrained by several challenges, the most important of which is the rise of insecticide resistance [39, 40]. Therefore, the need for complementary interventions to sustainably combat malaria and other mosquito-borne diseases and hence achieve elimination is crucial. There is a need for complementary interventions. House improvement provides a novel option since the transmission of malaria and several other mosquito-borne diseases predominantly occurs indoors [41]. It has been proven that when houses are fully screened, blocking the open eaves and tightening the doors and windows can provide full protection against mosquito bites [13, 21]. Also, a previous study reported that urbanisation has had a great impact on reducing the number of malaria mosquitoes [42] and that the need for house improvement is of important. VICOBA has the potential to provide platforms for community education on malaria and other health aspects. Interestingly, many studies indicate VICOBA’s great impact on livelihood [29, 30], and recommend integration with health services [33]. However, VICOBA has great potential for a self-sustaining financial system if taken into consideration for house improvements against malaria and other mosquito-borne diseases.

Findings from the present study showed the community’s need for housing improvement, as reported by Bofu *et al*., [25] for instance, participants reported that their houses had opened eaves, unscreened windows, and doors that were not well fitted. Also, they reported that their house’s walls were either plastered or unplastered; some houses had mud walls; and the roofs of their houses were either metal roofs or grass thatched roofs. The previous study found that houses with these characteristics allowed both malaria and non-malaria mosquitoes to enter the houses as well as rest inside them [24, 37]. For example, during FGD, participants also responded that in their villages, the situation with houses is not good since most of them are unfinished. Their villages have an irrigation system throughout the year that makes the mosquito density high and hence the risk of diseases. They also reported that not all houses need major improvements, which makes it possible to use the VICOBA platform to improve their houses. Participants responded that if the loans can be used for sending children to school and agricultural purposes, they can also be used to improve the houses. Since members have the chance to take loans more than once, they also have the chance to fulfil their needs in stages. For example, if a person takes a loan for agricultural purposes, he or she can take the first loan for leasing the farm, initial preparation, and sowing the seeds. After the first round of the loan, the second loan will be used for other purposes, like weeding the plants and putting down manure.

Moreover, participants reported that due to their housing conditions, the community is facing a high mosquito problem, and they have been bitten by mosquitoes many times while around their houses in the evenings and nights. Participants explained that mosquitoes use doors, windows, and open eaves to enter their houses. Unfortunately, the major protection mechanism reported was the bed net and few were using tropical repellent and insecticide spray while others didn’t have any protection. However, as evidenced by the previous study, the houses with open eaves and unscreened windows can have a high mosquito density inside, hence the increased of risk of getting malaria and other mosquito borne-diseases.

Respondents reported having different needs for improving their houses. Some need to block the open eaves, some need to screen the windows, some need to tighten the doors, and some need all three. The amount needed for improving houses will differ depending on the need of the house, and the borrowing will be in stages because, with the small amount that they borrow, they will not manage to fulfil the purposes with just one round of loans. Participants will be given loans depending on the amount needed for housing improvement; they will be supposed to save one-third of the amount that they want to borrow because that amount is usually used as collateral for the loan that the member borrows from the group. For example, if a participant wants 300,000 tsh, they will need to save 100,000 tsh. The repayment will depend on the borrower’s capacity to pay; usually, the borrower has to choose between paying within three months or six months, and the interest is 10% of the amount borrowed. Also, some groups agree to pay only interest every month, and loans will be paid at the end of the cycle. Also, in the group, they usually agree on the minimum and maximum amount of savings at every meeting. For example, groups might agree the minimum saving is 10,000 Tshs; during meetings, every member must come with that saving, and this is a must for those who are willing to save more than 10,000 Tshs.

This present study also revealed that a few community members had already been using loans from VICOBA to build houses. This result indicate that it is possible for group members to use loans from VICOBA for improving houses. It also shows the potential for ensuring the sustainable availability of funds for regular house improvements, which could benefit both the control of malaria and other vector-borne diseases predominantly transmitted indoors. However, in this present study, it was noted that group members have been saving and borrowing money from VICOBA for more than three years. They have been saving money every week, and each member borrows once within six months and repays the borrowed amount on a monthly basis, but some of the groups borrow and return within three months; in this case, this person can borrow four times within a year. The group lasts for twelve months, during which each member has to finish the loan. In this case, members are eligible to borrow money twice a year, depending on the agreement of the group members and on the duration of the loans. However, after one year, on the same day they close the year, they start another round of VICOBA. That’s why there are some groups that have had more than 3 to 4 years.

This study yielded evidence that, due to various reasons such as urgent personal expenses, school fees, ceremonies, unforeseen emergencies, or other financial pressures, members allocated borrowed funds to lower priority expenses, leaving insufficient funds to cover house improvements. Having a significant amount of money at hand might not always be the reality for community members looking to make housing improvements. Nevertheless, the option to borrow funds could enable them to gradually improve their houses. This can be achieved through strategizing and by prioritizing improvements at the primary mosquito entry points and gradually progressing to the less essential ones. This staged approach could align with their ongoing borrowing through VICOBA, allowing for a systematic and sustainable home improvement process.

This study has certain limitations as it relied on individual VICOBA members to complete the questionnaire, which may have resulted in biased responses to certain questions. For example, when asked whether they managed to repay the credited fund, the majority responded that they did, but this may not be entirely accurate. Also, the study did not assess the group leaders’ overall performance or management of the groups. This study also encourages more qualitative studies, which should be considered since the general idea of using VICOBA involves the social-economic behaviours of the VICOBA members. However, the observation of the whole process from the beginning of VICOBA to the closing of the round should also be considered in future studies to get the whole picture of what is happening in VICOBA groups. Additionally, the study was unable to establish a clear link between members’ income generation and their ability to repay the funds at this stage. Another limitation is the lack of involvement of government representatives and other stakeholders to gather their perspectives on the study. This study used focus group discussions (FGDs) as the primary method for collecting qualitative data. FGDs were deemed suitable for our research objectives as they allow for group dynamics and interactions, facilitating the exploration of shared experiences, perceptions, and opinions among community members. However, we recognize the importance of additional qualitative methods, such as in-depth interviews, to capture the viewpoints of specific individuals or key informants, including VICOBA leaders. This was another limitation of the study, as it did not use interviews.

## Conclusion

This study provides evidence regarding the potential of VICOBA as a financial tool to support the lives of poor communities in rural settings. The study shows the communities live in houses with poor conditions that allow mosquitoes indoors. All VICOBA members supported the idea of using the VICOBA platform for house improvement; however, there were gaps in community sensitization on the use of VICOBA for uplifting health interventions such as house improvement for mosquito control. Furthermore, engaging key stakeholders such as the ministry of health, the ministry of land, house, and settlement, the ministry of industry, the ministry of finance, researchers, and community members would harmonize the idea of house improvement for mosquito-borne disease control.

## Supporting information

S1 Data Dataset analysed for the findings of “housing characteristics, knowledge about malaria and its association with household characteristics and knowledge and willingness to use VICOBA for housing improvement and malaria control”. (XLSX)

## Abbreviations

ITNs: Insecticide-treated nets
IRS: Indoor Residual Spraying
VICOBA: Village Community Bank
FGD: Focused group discussion
SSA: Sub Saharan Africa
MRDT: Malaria Diagnostic test
